# Interrupted arch or extreme coarctation?

**DOI:** 10.1016/j.xjse.2026.100107

**Published:** 2026-02-25

**Authors:** Thierry Carrel

**Affiliations:** Department of Cardiac Surgery, University Hospital Basel, Basel, Switzerland

To the Editor:

**Figure:**
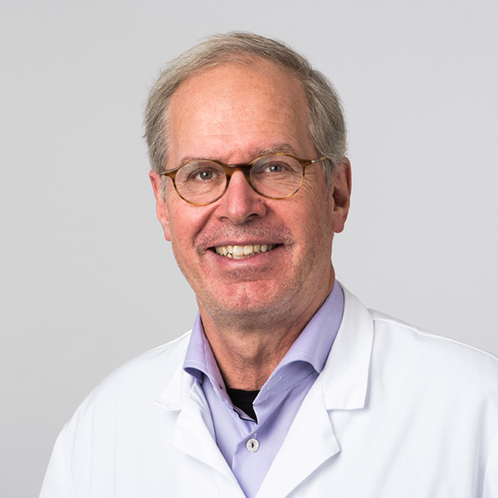

I read with interest the case report by Selek and colleagues^1^ and would like to congratulate the surgical team for the outstanding surgical result obtained in such a challenging case. When reading the history of the patient and reviewing the pre- and postoperative images, I believe that this was most probably a case of extreme coarctation rather than a true interruption of the aortic arch. Four features may speak in favor of this hypothesis:

• First, the distance between the proximal and distal “stumps” of the suspected interruption is extremely short and on the magnetic resonance imaging, there is no loss of continuity between the proximal and distal segments. It seems rather like a very narrowed membrane than a true interruption.
• Second: on the computed tomography scan, there is no difference in contrast between the proximal and distal parts of the interrupted aorta; this means that the contrast medium is flowing very quickly, not only through collaterals but most probably antegradely through the native but very narrowed aorta. In that sense, the present case most probably resulted from a gradual worsening of the undiagnosed aortic coarctation to a very tight “hourglass”-like stenosis that looked like a functional interruption.
• Third: a potential continuity was not explored with a catheter during angiography. Of course, this would not be reasonable in an acute situation, but in children, direct angiography still represents the best method to allow a clear differentiation between extremely critical stenosis (coarctation) and a true agenesia of a short segment (interruption) of the aortic arch
• Fourth: as well-pointed out by the authors, untreated interrupted aortic arch has an almost 100% rate of mortality during infancy, with survival over 17 years of age being absolutely exceptional.

The technique of the distal anastomosis is not well understood (“Because of the size mismatch between the graft and the native aorta, the distal anastomosis was left partially incomplete on the left anterolateral aspect”)^1^ and, in addition, it is not really clear at which level it was performed because the dissection extended into the innominate artery and the anastomosis was performed to the nondissected aorta (was the anastomosis performed at the level of the proximal arch, was the innominate artery reimplanted?)

Sometimes, when a large mismatch between the graft and the distal aorta is present, it is possible to adapt as much as possible this special feature and create a somewhat cone-shaped distal end of the graft. If not, the graft may be gently plicated to reduce its distal diameter.

Doing this, the extra-anatomic graft modification may then be connected very easily with the prosthetic graft of the ascending aorta through an end-to-side anastomotic technique, exactly like in an elective case of complex or multiple redo coarctation. This may by the way facilitate deairing of both grafts.

Further in the text, the authors describe that “Inspection of the ascending aorta revealed dissection involving all 3 aortic valve cusps, with an entry tear at the left coronary cusp.” The cusps of the aortic valve never dissect, but all 3 sinuses of Valsalva may be affected by the dissection.

I would have been careful when writing that “[E]nd-to-end anastomosis was not feasible in this case because of the long-segment interruption and the inelasticity of adult aortic tissue.” In general, without having tried to do so, it is not possible to claim that a direct anastomosis would not have been feasible because the gap between the proximal and distal segments of the aorta seems to be very short on those images available in the publication. In addition, the authors did not test the aortic tissue at this specific location (“inelasticity”).

Finally, there is probably a mistake with the reference when the authors write that “This hypothesis, supported by J. A. Elefteriades^2^ his review of adult IAA cases, suggests that some late-presenting patients may have acquired interruption rather than a true congenital defect.” However, the paper by Elefteriades^2^ does not include any information related to interrupted aortic arch.

## Conflict of Interest Statement

Dr Carrel is a Medical Advisor for Novostia AG and Swiss Cardio Technologies, a company of Abacus Medicine.

The *Journal* policy requires editors and reviewers to disclose conflicts of interest and to decline handling or reviewing manuscripts for which they may have a conflict of interest. The editors and reviewers of this article have no conflicts of interest.
